# Organization and training at national level of antimicrobial stewardship and infection control activities in Europe: an ESCMID cross-sectional survey

**DOI:** 10.1007/s10096-019-03648-2

**Published:** 2019-08-08

**Authors:** Alberto Enrico Maraolo, David S. Y. Ong, Cansu Cimen, Philip Howard, Diamantis P. Kofteridis, Jeroen Schouten, Nico T. Mutters, Céline Pulcini, on behalf of the ESGAP-EUCIC-TAE Working Group on AMS/IPC mapping in Europe, Arjan Harxhi, Elisabeth Presterl, Iris Zeller, Agnes Wechsler-Fördös, Akif Gurbanov, Stien Vandendriessche, Hilde Jansens, Tomi Kostyanev, Rossitza Vatcheva-Dobrevska, Maja Sabolić, Rok Civljak, Vera Vlahovic-Palcevski, Milan Trojanek, Maria Yiannitsarou, Constantinos Tsioutis, Kristina Obrink-Hansen, Bente Olesen, Karmen Jaaniso, Mari Ala-Houhala, Vincent Jarlier, Alexandre Bleibtreu, Thea-Christin Zapf, Winfred V. Kern, Frauke Mattner, Kyriakos Zaragkoulias, Athanassios Tsakris, Edit Hajdú, Gyula Prinz, Szabo Balint Gergely, Aaron Doherty, Kirsten Schaffer, Aoife Fleming, Khetam Hussein, Elena Carrara, Leonardo Pagani, Daniele Roberto Giacobbe, Albina Ponosheci-Bicaku, Lul Raka, Shaip Krasniqi, Alise Grāmatniece, Uga Dumpis, Rolanda Valinteliene, Tomas Kacergius, Viviane Knepper, Peter Zarb, Gertjan Wagenvoort, Andreas Voss, Per Espen Akselsen, Dag Berild, Joanna Kubiak, Aleksander Deptuła, Monika Wanke-Rytt, Filipa Sofia de Sousa Fernandes, Nuno Rocha-Pereira, Carlos Palos, Neda Milevska Kostova, Diana Gabriela Iacob, Oana Sandulescu, Roxana Filip, Aleksandra Barac, Vladimír Krčméry, Maja Plesko, Tatjana Lejko Zupanc, Bojana Beović, José Ramón Paño Pardo, Juan Pablo Horcajada, Zaira Palacios Baena, Thomas Tängdén, Anders Johansson, Caroline Rönnberg, Benedikt Huttner, Walter Zingg, Murat Akova, Önder Ergönül, Alison Holmes, Muge Cevik, Aidyn Salmanov

**Affiliations:** 1 Department of Clinical Medicine and Surgery, Section of Infectious Diseases, University of Naples “Federico II”, Naples, Italy; 2 Department of Medical Microbiology and Infection Control, Franciscus Gasthuis & Vlietland, Kleiweg 500, 3045 PM Rotterdam, The Netherlands; 3 Department of Epidemiology, Julius Center for Health Sciences and Primary Care, University Medical Center Utrecht, Utrecht University, Utrecht, The Netherlands; 4 Infectious Diseases and Clinical Microbiology Clinic, Ministry of Health, Ardahan Public Hospital, Ardahan, Turkey; 5 Leeds Teaching Hospitals NHS Trust, Leeds, UK; 6 Faculty of Medicine, Department of Internal Medicine, University Hospital of Heraklion and University of Crete, Heraklion, Crete Greece; 7 Scientific Center for Quality of Healthcare, IQ Healthcare, Radboud University Medical Center, Nijmegen, The Netherlands; 8 Institute for Infection Prevention and Hospital Epidemiology, Medical Centre, Faculty of Medicine, University of Freiburg, Freiburg, Germany; 9 APEMAC and Infectious Diseases Department, Université de Lorraine and CHRU-Nancy, Nancy, France

**Keywords:** Antimicrobial stewardship, Infection prevention and control, Clinical microbiology, Infectious diseases, Questionnaire

## Abstract

Antimicrobial stewardship (AMS) and Infection prevention and control (IPC) are two key complementary strategies that combat development and spread of antimicrobial resistance. The ESGAP (ESCMID Study Group for AMS), EUCIC (European Committee on Infection Control) and TAE (Trainee Association of ESCMID) investigated how AMS and IPC activities and training are organized, if present, at national level in Europe. From February 2018 to May 2018, an internet-based cross-sectional survey was conducted through a 36-item questionnaire, involving up to three selected respondents per country, from 38 European countries in total (including Israel), belonging to the ESGAP/EUCIC/TAE networks. All 38 countries participated with at least one respondent, and a total of 81 respondents. Education and involvement in AMS programmes were mandatory during the postgraduate training of clinical microbiology and infectious diseases specialists in up to one-third of countries. IPC was acknowledged as a specialty in 32% of countries. Only 32% of countries had both guidance and national requirements regarding AMS programmes, in contrast to 61% for IPC. Formal national staffing standards for AMS and IPC hospital-based activities were present in 24% and 63% of countries, respectively. The backgrounds of professionals responsible for AMS and IPC programmes varied tremendously between countries. The organization and training of AMS and IPC in Europe are heterogeneous and national requirements for activities are frequently lacking.

**Electronic supplementary material:**

The online version of this article (10.1007/s10096-019-03648-2) contains supplementary material, which is available to authorized users.

## Introduction

The epidemiology of antimicrobial resistance (AMR) presents a heterogeneous pattern worldwide [1]. This is the result of a complex interaction of factors, some of them beyond the reach of healthcare professionals such as antimicrobial use in agriculture [2]. The heterogeneous implementation of antimicrobial stewardship (AMS) and infection prevention and control (IPC) programmes in different countries also contributes to this variation [3–6]. However, little is known regarding the differences in organization and training of AMS and IPC activities between countries.

The purpose of this survey was to provide an overview of how AMS and IPC are integrated in the postgraduate training of ID and CM specialists and how hospital-based AMS and IPC activities are organized at national levels in European countries.

## Methods

### Setting and participants

The European Society of Clinical Microbiology and Infectious Diseases (ESCMID) Study Group for Antimicrobial StewardshiP (ESGAP), the European Committee on Infection Control (EUCIC) and the Trainee Association of ESCMID (TAE) performed a cross-sectional survey in Europe and Israel.

The 36-item online questionnaire (online_supplementary_file_1) was web-based, using SurveyMonkey® software (Palo Alto, CA, USA). Three representatives from each of the 38 countries, i.e. one respondent representing each group (ESGAP, EUCIC and TAE), were selected based on their expertise and experience in AMS and IPC activities. To have a comprehensive snapshot of activities at national level, we asked the respondents to be as representative as possible of what was occurring nationwide in their working country, even by asking other colleagues for help to answer specific questions if needed.

Participation was voluntary without any financial compensation. The purpose of the survey was clearly stated to participants before the survey started. Ethics approval was not necessary according to current regulations.

### Survey design and data analysis

The questionnaire was divided in three parts. The first addressed general topics such as the way CM, ID and IPC are organized as specialties and represented at national level through official societies or even through informal organizations when it comes to trainee associations. The second and third parts were focused on AMS and IPC hospital-based activities, respectively. The respondents were encouraged to add comments and further details (e.g. weblinks or references of official documents) for each question. Although the total number of items was 36, the true number of questions to be answered could be lower, because of some filter questions.

The questionnaire was open between 18 March and 31 May 2018. The representatives from each country were contacted through e-mail by a member of ESGAP, EUCIC or TAE, providing the weblink of the survey. Periodic reminders were sent in order reach the best possible response rate.

Once the survey was closed, data completeness and consistency were checked, comparing the replies of the different respondents from the same country. Subsequently, if necessary, respondents were contacted again to solve discrepancies by majority consensus or to clarify unclear replies. If available, the investigators also double checked the official documents provided by the national representatives.

## Results and discussion

### Participants, national societies and trainee associations

The overall response rate was 81/114 (71%) (eTable 1). In 18 (47%) countries, the survey was filled in by three respondents (one from each group ESGAP/EUCIC/TAE), in 7 (18%) countries, there were two respondents, and one respondent replied in 13 (34%) countries.

A national professional CM and ID society was only present in 23 (61%) and 31 (82%) countries, respectively (eTables 2 and 3). In 5 (13%) countries, CM/ID were officially represented by a joint society. Official IPC and CP societies were both present in 21 (55%) countries.

A formal association of trainees was present in 10 (26%), 12 (32%), 4 (11%), and 7 (18%) countries for CM, ID, IPC and CP, respectively.

### Organization and training requirements

The organization of CM, ID and IPC specialties and AMS/IPC postgraduate training was very heterogeneous (eTable 4). CM and ID were separated disciplines in 34 (89%) countries, but in two countries offered as dual training. In 26 (68%) countries, CM was a stand-alone specialty, whereas in 10 (26%), it was a sub-specialty of another discipline (mostly laboratory medicine). CM was mostly open to medical doctors only, but in some countries open to biologists and pharmacists. When ID was a subspecialty, it was generally in the framework of internal medicine. It is worrisome that in some countries, CM and ID were not recognized as specialty.

IPC was a stand-alone specialty in 7 (18%) countries and a subspecialty in 5 (13%) countries. Although in 3 countries, it was not framed as an official specialty, medical doctors could get a specific IPC qualification. In 26 (68%) countries, nurses had the possibility to obtain an IPC qualification after formal training.

Heterogenous findings and a quite variable duration of postgraduate training complicate the path to full recognition and accreditation of CM and ID professions in Europe [7]. The situation regarding IPC is even more fragmented. These results argue for an acceleration of the implementation of a standardized core curriculum for trainees in Europe [8, 9], thereby closing the current gaps among countries and harmonizing the quality of training [10, 11]. Furthermore, standardizing IPC training to become an IPC practitioner is likewise needed [7, 12, 13].

### AMS and IPC activities in hospitals

In half of the countries, AMS and IPC programmes were under the same hospital department, clinical leader or team (eTable 5). Routine interactions and collaborations between AMS and IPC professionals were seen in 28 (74%) of cases. The aim should be a daily and structured cooperation for a coordinated approach to tackle AMR [14].

**Fig. 1:**
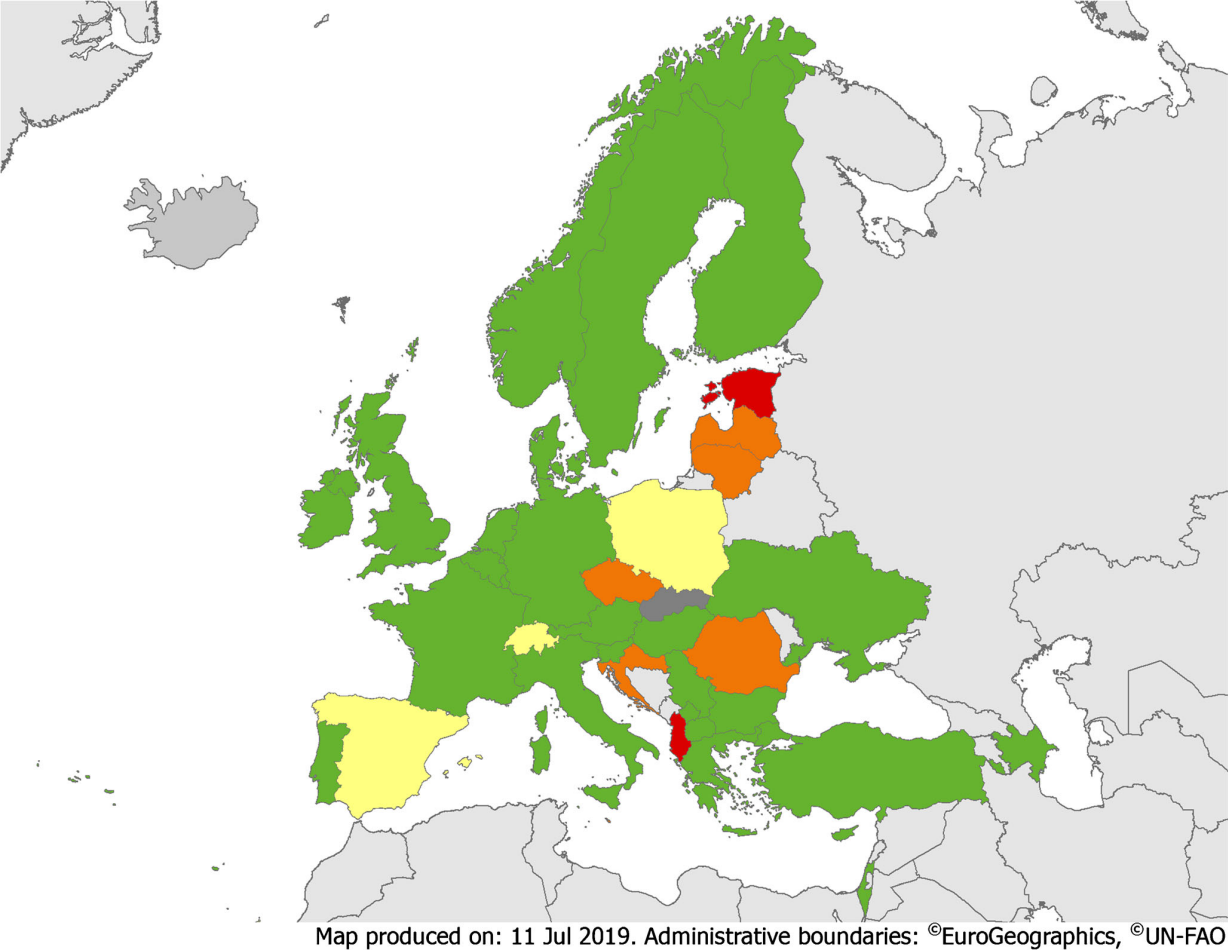
Overview of guidance or requirements on AMS and IPC implementation. Green = guidance or requirements on both AMS and IPC implementation. Yellow = guidance or requirements on AMS (but not on IPC implementation). Orange = guidance or requirements on IPC (but not on AMS implementation). Red = no guidance or requirements on AMS and IPC implementation. Dark grey = no data available

In 12 (32%) countries, there were guidance and national requirements on the implementation of AMS programmes; in 16 (42%), there was only general guidance (Fig. 1, eTable 6). These numbers are in line with a recently published survey [15], and different from IPC, where guidance and national requirements existed in nearly two-thirds of countries [16].

**Fig. 2:**
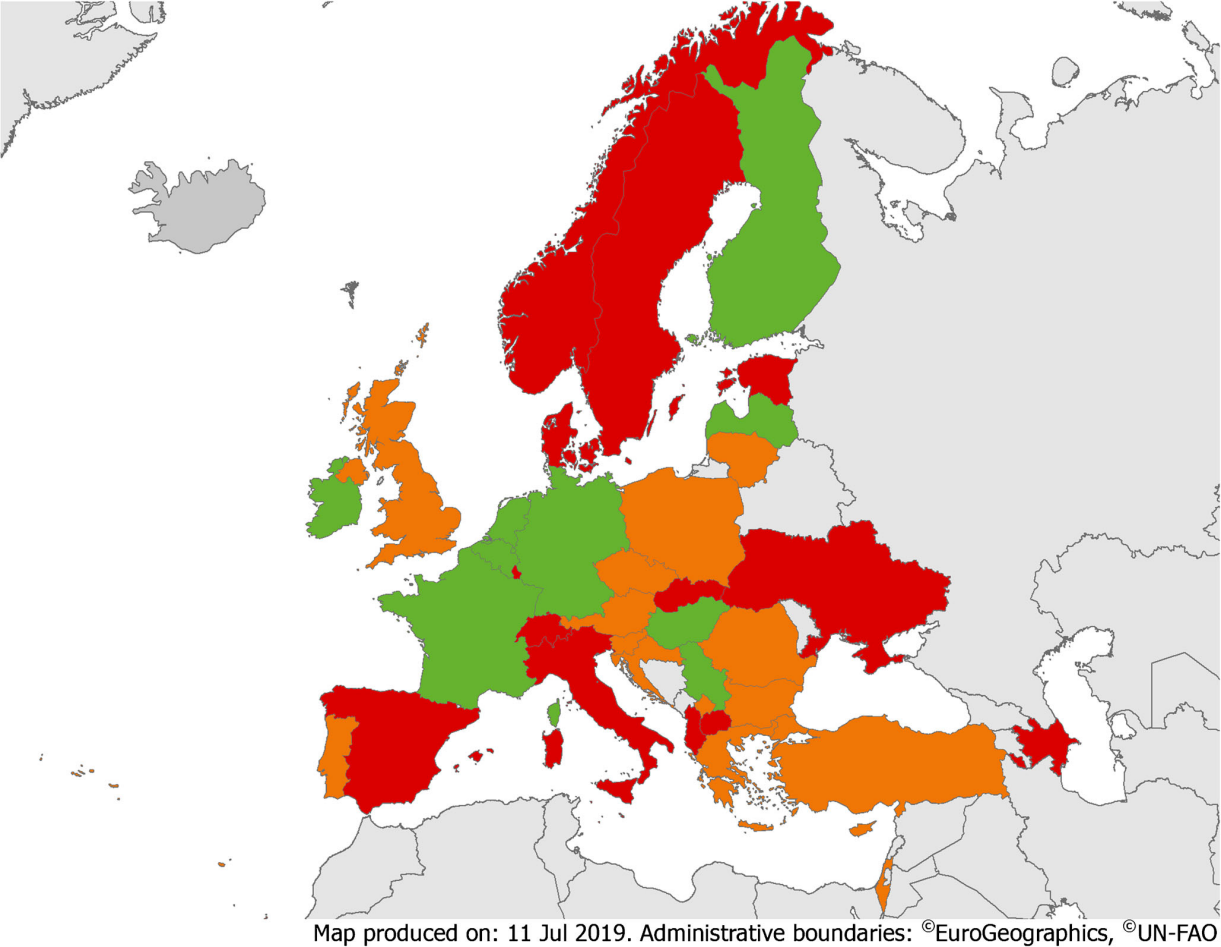
Overview of national staffing standards for AMS and IPC teams. Green = staffing standards for both AMS and IPC teams. Orange = staffing standards for IPC teams (but not for AMS teams). Red = no staffing standard for AMS and IPC teams

Formal national staffing standards (i.e. number of professionals per number of acute care beds) for AMS hospital-based activities existed in 9 (24%) countries (Fig. 2), which is in accordance with a previous survey [17]. Having staffing standards and proper funding to support AMS activities is crucial and has been included in global AMS core elements [14]. However, only 3 (8%) countries had official national requirements concerning formal training to become qualified as an AMS team member (eTable 7).

Guidance and national requirements on how to implement an IPC programme were present in 23 (61%) countries, and 24 (63%) had national staffing standards for IPC hospital-based activities (Figs. 1 and 2, eTable 8, eTable 9). A recent review recommended at least one FTE nurse per up to 250 beds and a dedicated physician [18]. Only 13 (34%) countries had national requirements regarding postgraduate training to become an IPC team member.

### Education during postgraduate training

**Fig. 3:**
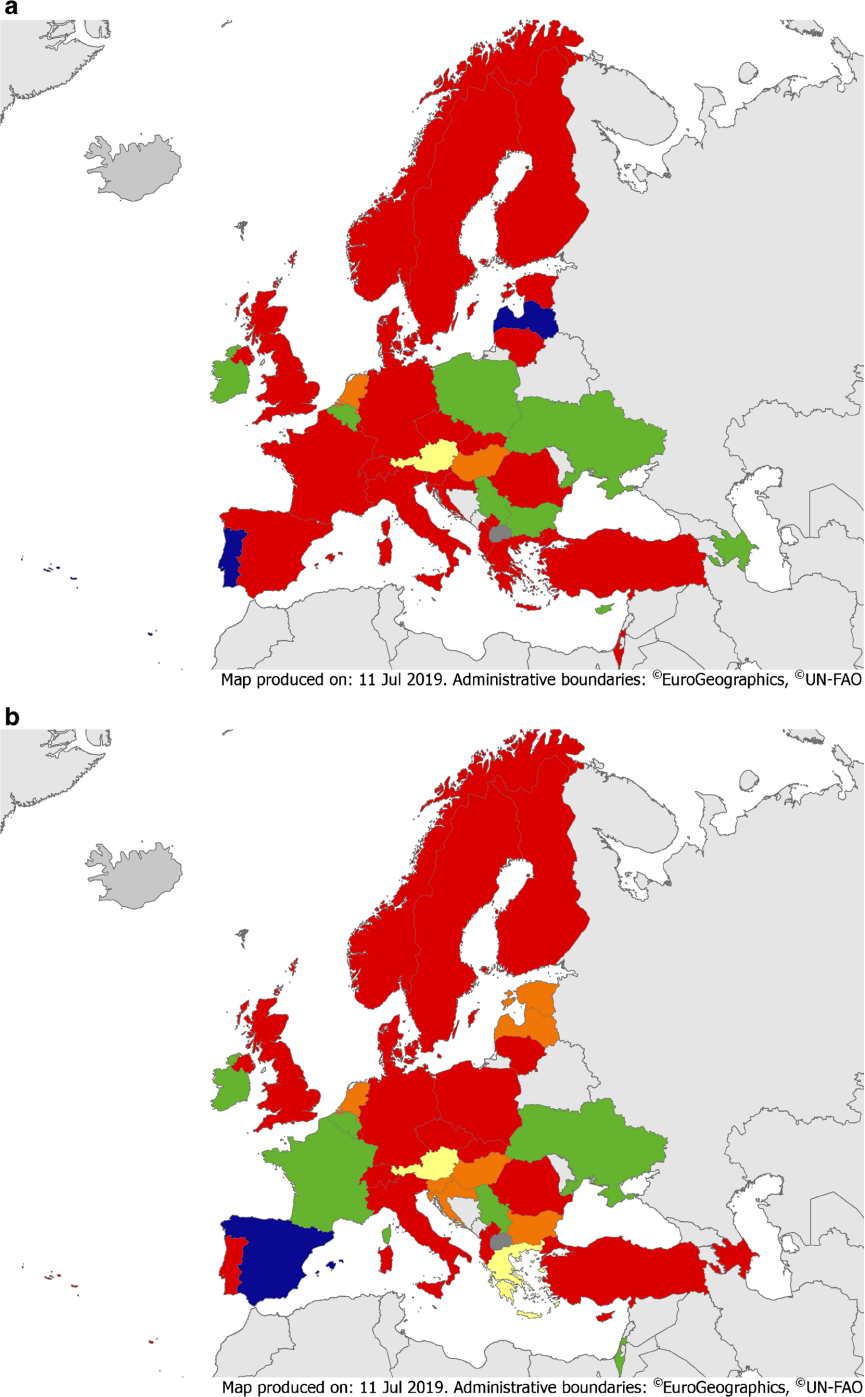
**a** Educational requirements on AMS for CM trainees. Green = mandatory formal training on AMS implementation and involvement in AMS activity during training. Yellow = mandatory formal training on AMS implementation (but no involvement in AMS activity during training). Orange = mandatory involvement in AMS activity during training (but no formal training on AMS implementation). Red = no mandatory formal training on AMS implementation or involvement in AMS activity during training. Blue = not applicable. Dark grey = no data available. **b** Educational requirements on AMS for ID trainees. Green = mandatory formal training on AMS implementation and involvement in AMS activity during training. Yellow = mandatory formal training on AMS implementation (but no involvement in AMS activity during training). Orange = mandatory involvement in AMS activity during training (but no formal training on AMS implementation). Red = no mandatory formal training on AMS implementation or involvement in AMS activity during training. Blue = not applicable. Dark grey = no data available

Mandatory formal training on how to implement an AMS programme was reported in 9/36 (25%), 8/37 (22%) and 5/15 (33%) countries for CM, ID and IPC specialties, respectively (Fig. 3a, b, eTable 10). The proportion of mandatory involvement in AMS activities during postgraduate training was 11/36 (31%), 13/37 (35%) and 6/15 (40%), respectively. Official training requirements to become an AMS team member were lacking in most countries, even though appropriate training is part of recently validated and globally relevant core elements [14]. Ideally, basic training in generic competencies for all prescribers regarding antibiotic use and stewardship should be delivered to all healthcare professionals [19].

**Fig. 4:**
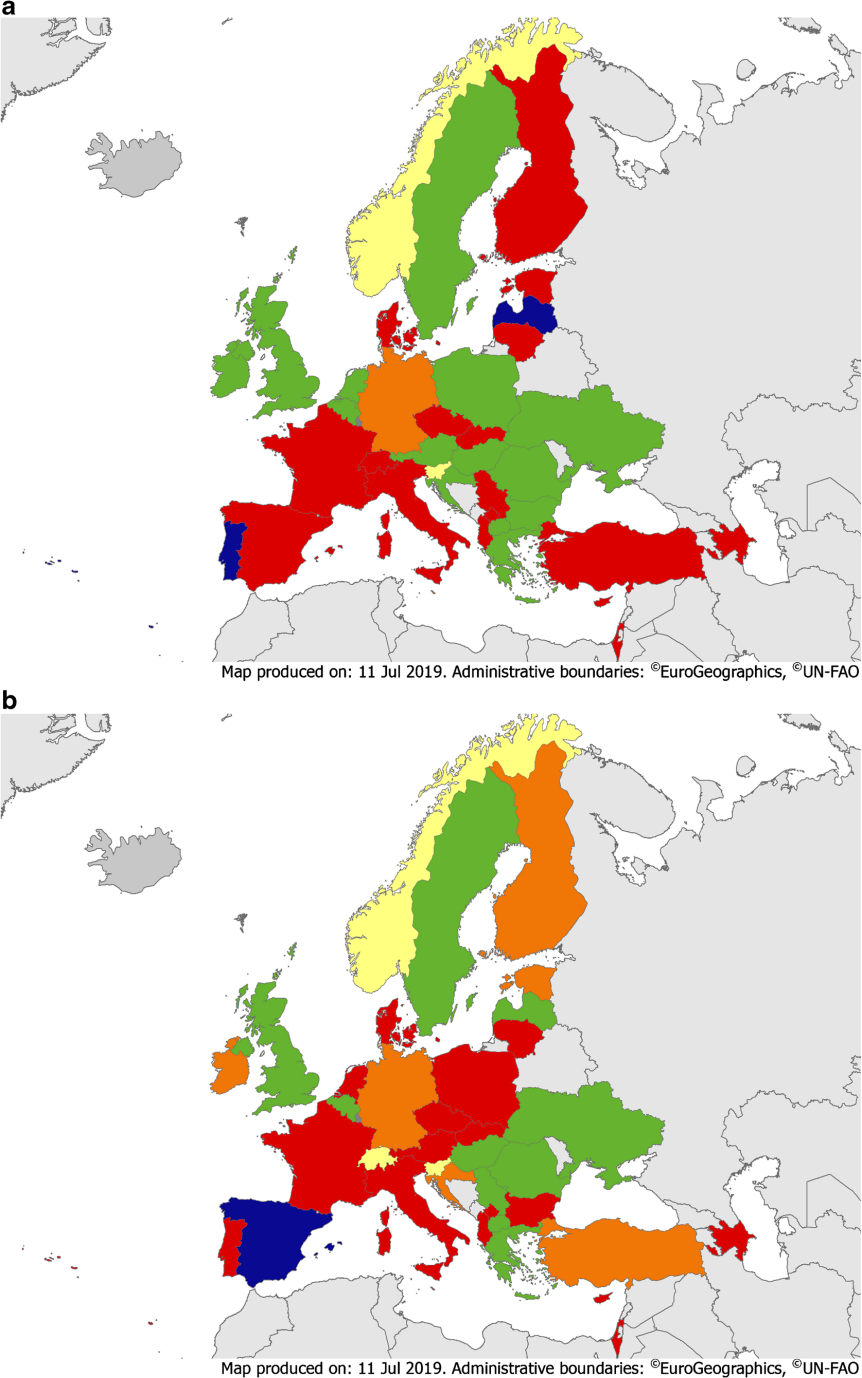
**a** Educational requirements on IPC for CM trainees. Green = mandatory formal training on IPC implementation and involvement in IPC activity during training. Yellow = mandatory formal training on IPC implementation (but no involvement in IPC activity during training) = Yellow Orange = mandatory involvement in IPC activity during training (but no formal training on IPC implementation). Red = no mandatory formal training on IPC implementation or involvement in IPC activity during training. Blue = not applicable. Dark grey = no data available. **b** Educational requirements on IPC for CM trainees. Green = mandatory formal training on IPC implementation and involvement in IPC activity during training. Yellow = mandatory formal training on IPC implementation (but no involvement in IPC activity during training) = Yellow Orange = mandatory involvement in IPC activity during training (but no formal training on IPC implementation). Red = no mandatory formal training on IPC implementation or involvement in IPC activity during training. Blue = not applicable. Dark grey = no data available

Mandatory official training on IPC implementation was reported in 16/36 (44%), 13/37 (35%) and 10/15 (67%) countries for CM, ID and IPC specialties, respectively (Fig. 4a, b, eTable 10). Furthermore, involvement in IPC activities during training for CM, ID and IPC was mandatory in 16/36 (44%), 16/37 (43%) and 13/15 (87%) countries, respectively. These low numbers are possibly linked to the lack of official acknowledgment of core competencies for IPC activities at national level [20], despite the availability of a well-defined set of requirements for European professionals [21].

### Professionals responsible for conducting AMS and IPC activities

CM specialists were involved in 24/36 (67%) and 22/37 (59%) countries with regard to AMS and IPC daily hospital-based activities, respectively (eTables 11 and 12). ID specialists were involved in 30/37 (81%) and 24/37 (65%) countries for AMS and IPC, respectively. CP were involved in AMS programmes in 25/38 (66%) countries and in IPC activities in 4/38 (11%). Lastly, nurses were involved in AMS and IPC activities in 8/38 (21%) and 32/38 (84%) countries, respectively.

### Limitations of the study

This study has limitations. First, only up to three representatives were consulted for each country and, in some instances, responses came from just one or two participants. Although participants were asked to provide reference documents to corroborate their answers, this information was not always available. Second, healthcare is organized at regional level in some countries, and the national situation reported here might not always reflect specific regional areas.

## Conclusion

This survey demonstrates substantial heterogeneity and large room for improvement in AMS and IPC activities in European countries, both from an educational and an organizational perspective. Standardization and constant improvement of these activities is imperative in the light of the recent and alarming data regarding attributable deaths and disability related to AMR in Europe [22].

## Electronic supplementary material

ESM 1 (PDF 273 kb)

ESM 2 (PDF 665 kb)
