# Supplementary material for: Organization and training at national level of antimicrobial stewardship and infection control activities in Europe: an ESCMID cross-sectional survey
Source: Eur J Clin Microbiol Infect Dis. 2019 Aug 8;38(11):2061–8. doi: 10.1007/s10096-019-03648-2 (PMC6800847; doi:10.1007/s10096-019-03648-2)
Supplement: Supplementary file 1 — (PDF 273 kb) [file 10096_2019_3648_MOESM1_ESM.pdf]

General-I

* 1. What is your name? (please note that all data will be completely anonymized as for analysis and reporting)

* 2. In which country do you work?

* 3. What is your specialty? (please select all that apply)

- [ ] Clinical Microbiology
- [ ] Infectious Diseases
- [ ] Other (please specify)

General-II

* 4. Is there a national professional society in the country where you work for? (please select all that apply)

- [ ] Clinical Microbiology (CM)
- [ ] Infectious Diseases (ID)
- [ ] Infection Prevention and Control (IPC)
- [ ] Clinical Microbiology and Infectious Diseases (together)
- [ ] A society combining Infection Prevention and Control with Clinical Microbiology and/or Infectious Diseases
- [ ] Hospital-based Clinical Pharmacists (hospital = inpatient setting, public or private)
- [ ] Infectious Diseases/Antimicrobial Stewardship (AMS) hospital-based Clinical Pharmacists

Please provide web adresses of societies
Please indicate if IPC or Clinical Pharmacy are part/subgroups of major organizations.

* 5. Is there a formal national trainee association/group for the following specialties?

	Yes/No/I do not know/Not applicable
CM	
ID	
Infection Prevention and Control	
Hospital-based Clinical Pharmacy	

Please leave a comment if necessary

* 6. Is there an informal national trainee association/group (e.g., a social network group) for the following specialties?

	Yes/No/I do not know/Not applicable
CM	
ID	
Infection Prevention and Control	
Hospital-based Clinical Pharmacy	

Please leave a comment if necessary

General-III

* 7. Are CM and ID separated specialties in the country where you work?

- Yes
- No
- I do not know
- Not applicable

Please leave a comment if necessary

* 8. How is ID organized as specialty in the country where you work?

- It is a stand-alone specialty (e.g., a separated specialty from all other disciplines)
- It is a sub-specialty (e.g., you need to specialize first in internal medicine)
- None of these
- Not applicable

Other (please specify)

General-IV

* 9. If ID is stand-alone specialty, is it open to medical doctors (MD) only or other professions/backgrounds as well?

- ◯ It is open to MD only
- ◯ It is open to other professions/backgrounds than MD as well
- ◯ Not applicable

Please specify the other professions/backgrounds

General-V

* 10. If ID is a sub-specialty, please specify which professions/backgrounds (e.g., MD, PharmD) and specialties it is opened to.

Profession(s)/Background(s)

Specialty/specialties

General VI

* 11. How is CM organized as specialty in the country where you work?

- It is a stand-alone specialty (e.g., a separated specialty from all other disciplines)
- It is a sub-specialty (e.g., you need to specialize first in medical biology)
- None of these
- Not applicable

Other (please specify)

General-VII

* 12. If CM is a stand-alone specialty, is it open to MD only or other professions/backgrounds as well?

- ◯ It is open to MD only
- ◯ It is open to other professions/backgrounds than MD as well
- ◯ Not applicable

Please specify the other backgrounds

General-VIII

* 13. If CM is a sub-specialty, please specify which professions/backgrounds (e.g., MD, PharmD) and specialties it is opened to.

Profession(s)/Background(s)

Specialty/specialties

General-IX

* 14. Do hospital-based CM usually do clinical rounds on hospital wards?

- ○ Yes
- ○ No
- ○ I do not know
- ○ Not applicable

Please leave a comment if necessary

General-X

* 15. How is IPC organized as specialty in the country where you work? (please note that this question does not refer to nurses)

- ◯ It is a stand-alone specialty (e.g., a separated specialty from all other disciplines)
- ◯ It is a sub-specialty (e.g., you need to specialize first in infectious diseases or clinical microbiology)
- ◯ None of these
- ◯ Not applicable

Other (please specify)

General-XI

* 16. If IPC is a stand-alone specialty, is it opened to MD only or to other professions/backgrounds as well (e.g. PharmD)? (please note that this question does not refer to nurses)

- ◯ It is open to MD only
- ◯ It is open to other professions/backgrounds than MD as well
- ◯ Not applicable

Please specify the other backgrounds

General-XII

* 17. If IPC is a sub-specialty, please specify which professions/backgrounds (e.g., MD, PharmD) and specialties it is opened to.

Profession(s)/Background(s)

Specialty/specialties

General-XIII

* 18. Do IPC specialists usually do clinical rounds on hospital wards? (for example - visit wards for outbreaks or routine healthcare-acquired infections rounds like *C.difficile*; please note that this question does not refer to nurses)

- ◯ Yes
- ◯ No
- ◯ I do not know
- ◯ Not applicable

Please leave a comment if necessary

* 19. Can nurses specialize in IPC in your working country (i.e. by getting formal training and a certification in IPC)?

- ◯ Yes
- ◯ No
- ◯ I do not know

Please leave a comment if necessary

General-XIV

* 20. How many years of postgraduate training are required for the following specialties? (please note that this question refers only to MD)

	Years/I do not know/Not applicable
CM specialists	
ID specialists	
IPC specialists	

Please leave a comment if necessary

* 21. Do they have mandatory clinical rotations? (please note that this question refers only to MD)

	Yes/No/I do not know/Not applicable
CM trainees	
ID trainees	
IPC trainees	

(please give details: number of months and type of department)

* 22. Do ID / CM work exclusively in hospitals? (please note that this question refers only to MD)

- ◯ Yes for both
- ◯ Yes for ID only
- ◯ Yes for CM only
- ◯ No
- ◯ I do not know
- ◯ Not applicable

Please leave a comment if necessary

General-XVI

* 23. Do AMS and IPC activities usually fall under the same hospital department / same clinical leader / same team?

- ◯ Yes
- ◯ No
- ◯ I do not know
- ◯ Not applicable

If yes, please indicate the name of team/group (e.g. AMS team, Sepsis team, Infection team...)

* 24. Do hospital-based professionals in AMS usually closely interact/collaborate with professionals in IPC in your working country?

- ◯ Yes
- ◯ No
- ◯ I do not know
- ◯ Not applicable

Please comment on the way they collaborate, or on the reasons explaining why the collaboration does not take place

AMS-I

* 25. Are there national guidance or requirements on how to implement an AMS programme in hospitals in your working country?

- ◯ Yes, both guidance and requirements exists
- ◯ Guidance exists, but no requirements
- ◯ Both guidance and requirements are missing
- ◯ I do not know
- ◯ Other (please specify below)
- ◯ Not applicable

If guidance/requirements exist, please provide web references.
Please leave further comments if necessary.

* 26. During their postgraduate training, do the following categories of trainees receive mandatory formal training on how to implement an AMS programme? (please note that this questions refers only to MD)

	Yes/No/I do not know/Not applicable
CM	
ID	
IPC	

If yes, please give details (including average number of hours)

* 27. During their postgraduate training, do the following categories of trainees need to participate in mandatory clinical rotations where they are involved in the activities of the hospital AMS programme? (please note that this questions refers only to MD)

	Yes/No/I do not know/Not applicable
CM	
ID	
IPC	

If yes, please give details (how long and what kind of tasks and responsibilities they are allowed to do)

AMS-II

* 28. Which professionals are usually responsible for conducting hospital AMS activities on a daily basis in your working country?
(please select all that apply)

- [ ] CM
- [ ] ID
- [ ] Pharmacist
- [ ] Nurse
- [ ] Other
- [ ] I do not know
- [ ] Not applicable

If other, please give details

* 29. Do you have national staffing standards for AMS hospital-based activities (e.g one full-time equivalent [FTE] ID/CM + one FTE pharmacist per 1000 acute care beds for the AMS team)?

- Yes
- No
- I do not know
- Not applicable

If yes, please provide details and web references if available

* 30. Are there national requirements for a specific formal postgraduate AMS training for specialists to become an AMS team member? (please note that this questions refers only to MD)

- Yes
- No
- I do not know
- Not applicable

If yes, please provide details (including number of hours and web references, if available)

IPC-I

* 31. Are there national guidance or requirements on how to implement an IPC programme in hospitals in your working country?

- ◯ Yes, both guidance and requirement exists
- ◯ Guidance exists, but no requirements
- ◯ Both guidance and requirements are missing
- ◯ I do not know
- ◯ Other (please specify below)
- ◯ Not applicable

If guidance/requirements exist, please provide web references.
Please leave further comments if necessary.

* 32. During their postgraduate training, do the following categories of trainees receive mandatory formal training on how to implement an IPC programme? (please note that this question refers only to MD)

	Yes/No/I do not know/Not applicable
CM	
ID	
IPC	

If yes, please give details (including average number of hours)

* 33. During their postgraduate training, do the following categories of trainees need to participate in mandatory clinical rotations where they are involved in the activities of the hospital IPC programme? (Please note that this question refers only to MD)

	Yes/No/I do not know/Not applicable
CM	
ID	
IPC	

If yes, please give details (how long and what kind of tasks and responsibilities they are allowed to do)

IPC-II

* 34. Which professionals are usually responsible for conducting hospital IPC activities on a daily basis in your working country?
(please select all that apply)

- [ ] CM
- [ ] ID
- [ ] Pharmacist
- [ ] Nurse
- [ ] Other
- [ ] I do not know
- [ ] Not applicable

If other, please specify.

* 35. Do you have national staffing standards for IPC hospital-based activities (e.g one full-time equivalent [FTE] ID/CM + one FTE IPC nurse per 1000 acute care beds for the IPC team)?

- ( ) Yes
- ( ) No
- ( ) I do not know
- ( ) Not applicable

If yes, please provide details and web references if available

* 36. Are there national requirements for a specific formal postgraduate IPC training for specialists to become an IPC team member? (please note that this question refers only to MD)

- ◯ Yes
- ◯ No
- ◯ I do not know
- ◯ Not applicable

If yes, please provide details (including number of hours and web references, if available)
