# Supplementary material for: Organization and training at national level of antimicrobial stewardship and infection control activities in Europe: an ESCMID cross-sectional survey
Source: Eur J Clin Microbiol Infect Dis. 2019 Aug 8;38(11):2061–8. doi: 10.1007/s10096-019-03648-2 (PMC6800847; doi:10.1007/s10096-019-03648-2)
Supplement: Supplementary file 2 — (PDF 665 kb) [file 10096_2019_3648_MOESM2_ESM.pdf]

**eTable 1. Number of participants per country**

| Country | Number |
|---|---|
| Albania | 1 |
| Austria | 3 |
| Azerbaijan | 1 |
| Belgium | 3 |
| Bulgaria | 1 |
| Croatia | 3 |
| Czech Republic | 1 |
| Cyprus | 2 |
| Denmark | 2 |
| Estonia | 1 |
| Finland | 1 |
| France | 3 |
| Germany | 3 |
| Greece | 3 |
| Hungary | 3 |
| Ireland | 3 |
| Israel | 1 |
| Italy | 3 |
| Kosovo | 3 |
| Latvia | 3 |
| Lithuania | 2 |
| Luxembourg | 1 |
| Malta | 1 |
| Netherlands | 2 |
| Norway | 2 |
| Poland | 3 |
| Portugal | 3 |
| Republic of Northern Macedonia | 1 |
| Romania | 3 |
| Serbia | 1 |
| Slovakia | 1 |
| Slovenia | 2 |
| Spain | 3 |
| Sweden | 3 |
| Switzerland | 2 |
| Turkey | 3 |
| Ukraine | 1 |
| United Kingdom | 3 |

**eTable 2. Presence of national professional societies and trainee associations for Clinical Microbiology, Infectious Diseases, Infection Prevention and Control, and Clinical Pharmacy, in the 38 surveyed European countries**

| Country | National professional societies | | | | | Formal and/or informal trainee associations | | | |
|---|---|---|---|---|---|---|---|---|---|
| ***Category*** | **CM** | **ID** | **IPC** | **CP** | **Other** | **CM** | **ID** | **IPC** | **CP** |
| Albania | X | X | | | | No | Only informal | No | No |
| Austria | | X | | X | CM+IPC | Only informal | No | No | Both |
| Azerbaijan | X | | | | | Yes, formal | Yes, formal | No | No |
| Belgium | | | X | X | CM+ID | Yes, formal | No | No | No |
| Bulgaria | X | X | X | X | | No | No | No | No |
| Croatia | X | X | | | | No | Both | No | Yes, formal |
| Czech Republic | X | X | X | | | Both | No | No | No |
| Cyprus | X | X | X | | | Only informal | No | Only informal | No |
| Denmark | X | X | X | X | | No | Only informal | No | No |
| Estonia | | X | | X | | No | No | No | No |
| Finland | X | X | X | | | No | Only informal | No | No |
| France | X | X | X | X | | Only informal | Both | No | No |
| Germany | | X | | X | CM+IPC | No | Both | No | No |
| Greece | X | X | X | | | Only informal | Yes, formal | Only informal | Only informal |
| Hungary | | | X | X | CM+ID | Both | Both | Both | Yes, formal |
| Ireland | X | X | X | X | | Both | Both | Yes, formal | Both |
| Israel | | X | | | | No | Both | Only informal | No |
| Italy | X | X | X | X | | No | No | No | No |
| Kosovo | X | X | | | | No | No | No | No |
| Latvia | | X | | | | No | No | No | No |
| Lithuania | | X | | X | | No | No | No | No |
| Luxembourg | | X | X | X | | Only informal | No | No | Both |
| Malta | | | | X | | Only informal | Only informal | No | Both |
| Netherlands | X | | X | X | | Both | No | No | Yes, formal |
| Norway | X | X | X | X | | No | No | No | Only informal |
| Poland | X | X | | | | No | No | No | No |
| Portugal | | | | | CM+ID | No | No | No | No |
| Republic of Northern Macedonia | | X | X | X | | No | Only informal | Only informal | No |
| Romania | X | X | | | CM+ID | No | No | No | Only informal |
| Serbia | X | X | | | | No | No | No | No |
| Slovakia | | X | | | | Yes, formal | Yes, formal | No | No |
| Slovenia | | X | | | | No | Only informal | No | No |
| Spain | | | X | X | CM+ID | No | No | No | No |
| Sweden | X | X | X | X | | Yes, formal | Yes, formal | Only informal | No |
| Switzerland | X | X | X | X | | No | No | No | No |
| Turkey | X | X | X | | | Yes, formal | Yes, formal | No | No |
| Ukraine | X | X | X | X | | No | Only informal | Yes, formal | No |
| United Kingdom | X | X | X | X | | Both | Both | Both | Only informal |

CM, Clinical Microbiology; ID, Infectious Diseases; IPC, Infection Prevention and Control; CP, Clinical Pharmacy.

**eTable 3. Web addresses of national societies of specialties involved in AMS/IPC activities**

| Country | URL | | | | |
|---|---|---|---|---|---|
| *Category* | *CM* | *ID* | *IPC* | *CP* | *Other* |
| Albania | NP | www.shshi.al | NP | NP | |
| Austria | | http://www.oegit.eu | | www.kh-pharmazie.at; https://oephg.eu/ | http://www.oegach.at http://www.oegkh.ac.at (CM+IPC) |
| Azerbaijan | NP | NP | NP | NP | |
| Belgium | | | http://www.belgianinfectioncontrolsociety.be/v2/home/startpage/ | http://www.hospitalpharmacistbelgium.eu | http://www.bvikm.org/ (CM+ID) |
| Bulgaria | www.bam-bg.net | http://bsid-bg.org/ | www.bulnoso.org | NP | |
| Croatia | http://www.hdkm.hr | http://hdib.hr | NP | NP | www.iskra.bfm.hr |
| Czech Republic | www.sem-cls.cz | www.infekce.cz | www.sneh.cz | NP | |
| Cyprus | NP | NP | NP | NP | |
| Denmark | www.dskm.dk | www.infemed.dk | NP | NP | |
| Estonia | NP | www.esid.com | NP | NP | |
| Finland | https://kliinisetmikrobiologit.fi | https://infektiolaakarit.yhdistysavain.fi | www.sshy.fi | NP | |
| France | http://www.sfm-microbiologie.org | http://www.infectiologie.com | https://sf2h.net | http://sfpc.eu/fr | |
| Germany | | www.dgi-net.de | | www.adka.de | www.dghm.org (CM+IPC) www.antibiotic-stewardship.de www.krankenhaushygiene.de |
| Greece | https://hms.org.gr/en/ | www.loimoxeis.gr | www.eeel.gr | NP | |
| Hungary | | | www.infekciokontroll.hu | https://www.mgyt-kgysz.hu/ | www.infektologia.hu (CM+ID) |
| Ireland | http://www.iscm.ie/website/ | https://www.idsociety.ie | www.ipcireland.ie | https://hpai.ie/ | http://www.hpsc.ie/ |
| Israel | NP | NP | NP | NP | |
| Italy | http://www.amcli.it/ | http://www.simit.org | http://www.societaitalianaigiene.org | http://www.sifoweb.it | www.simpios.it |
| Kosovo | NP | NP | NP | NP | |
| Latvia | NP | NP | NP | NP | |
| Lithuania | NP | https://www.lid.lt/ | NP | https://www.lkfd.lt/ | |
| Luxembourg | NP | NP | NP | NP | www.conseil-scientifique.lu |
| Malta | NP | NP | NP | http://www.mahp.org.mt | |
| Netherlands | https://www.nvmm.nl/ | www.infectieziekten.org | https://www.vhig.nl/ | https://nvza.nl/ | https://www.rivm.nl/ |
| Norway | https://legeforeningen.no/Fagmed/Norsk-forening-for-medisinsk-mikrobiologi/ | http://legeforeningen.no/Fagmed/Norsk-forening-for-infeksjonsmedisin/ | http://www.smittevernforum.no/ | www.sykehusfarmasi.org | |
| Poland | http://www.microbiology.pl | NP | NP | NP | http://www.antybiotyki.edu.pl |
| Portugal | | | https://www.dgs.pt/ | NP | http://spdimc.org (CM+ID) |
| Republic of Northern Macedonia | NP | http://www.infektivnaklinika.mk/Zdruzenie/Zazdruzenieto.htm | http://www.mzkihi.mk/index-en | http://www.mfd.org.mk | |
| Romania | http://www.srm.ro | http://www.infectiologie.ro | http://www.srepi.ro | NP | |
| Serbia | http://www.mikrobiologija.org | NP | NP | NP | |
| Slovakia | NP | https://www.infektologia.sk | NP | NP | |
| Slovenia | http://www.imi.si | http://www.szd.si | NP | http://www.sfd.si | http://www.szpz.info |
| Spain | | | http://www.sempsph.com http://www.seimc.org | https://www.sefh.es | www.seimc.org (CM+ID) |
| Sweden | http://www.mikrobiologi.net | www.infektion.net | https://sfvh.se/ https://slf.se/svenska-hygienlakarforeningen/ | https://www.apotekarsocieteten.se | |
| Switzerland | https://www.swissmicrobiology.ch | http://www.sginf.ch | https://www.sgsh.ch | NP | |
| Turkey | https://www.klimud.org | http://www.enfeksiyon.org.tr | http://www.hider.org.tr | NP | https://www.klimik.org.tr (CM+ID) |

| | | | | | |
|---|---|---|---|---|---|
| Ukraine | NP | NP | http://www.infectioncontrol.org.ua | | |
| United Kingdom | https://microbiologysociety.org | https://www.britishinfection.org | http://www.infectioncontrol.org.ua; https://www.ips.uk.net | http://ukclinicalpharmacy.org | www.bsac.org.uk; https://www.his.org.uk |

AMS, Antimicrobial Stewardship; IPC, Infection Prevention and Control; URL, Uniform Resource Locator; CM, Clinical Microbiology; ID, Infectious Diseases; CP, Clinical Pharmacy; NP, not provided (or absent)

**eTable 4. Organization of CM/ID/IPC specialties (official university track) across Europe**

| Country | General | Clinical Microbiology | | | | Infectious Diseases | | | | Infection Prevention and Control | | | | |
|---|---|---|---|---|---|---|---|---|---|---|---|---|---|---|
| | CM and ID as separated specialties | Type of specialty | Open only to MD | Years of PG training | Mandatory clinical rotations during PG training | Type of specialty | Open only to MD | Years of PG training | Mandatory clinical rotations during PG training | Type of specialty[a] | Open only to MD | Years of PG training | Mandatory clinical rotations during PG training | Possibility for nurses to specialize in IPC |
| Albania | Yes | SA | Yes | 4 | NP | SA | Yes | 4 | Yes | NA | NA | NA | NA | No |
| Austria | Yes | SA | Yes | 6 | Yes | SB | Yes | 6 | Yes | SA | Yes | 6 | Yes | Yes |
| Azerbaijan | Yes | SB | Yes | 2 | Yes | SB | Yes | 4 | Yes | SA | Yes | NP | No | No |
| Belgium | Yes | SB | No | 5 | Yes | SB | Yes | 5 | Yes | SB | No | 1 | Yes | Yes |
| Bulgaria | Yes | SA | Yes | 4 | Yes | SA | Yes | 4 | Yes | SB | No | 2 | Yes | Yes |
| Croatia | Yes | SA | Yes | 5 | Yes | SA | Yes | 5 | Yes | SB | Yes | NP | Yes | Yes |
| Czech Republic | Yes | SA | No | 5 | Yes | SA | Yes | 4 | Yes | SA | No | 4 | Yes | Yes |
| Cyprus | Yes | SB | Yes | 5 | Yes | SB | Yes | 7 | Yes | NA | NA | NA | NA | Yes |
| Denmark | Yes | SA | Yes | 6 | Yes | SA | Yes | 6 | Yes | NA | NA | NA | NA | Yes |
| Estonia | Yes | SB | Yes | 4 | Yes | SA | Yes | 4 | Yes | Other | Yes | 4 | NA | No |
| Finland | Yes | SA | No | 5 | Yes | SA | Yes | 6 | Yes | Other | Yes | 4 | Yes | Yes |
| France | Yes | SB | No | 4 | Yes | SA | Yes | 5 | Yes | Other | No | 5 | Yes | No |
| Germany | Yes | SA | Yes | 5 | Yes | SB | Yes | 7 | Yes | SB | Yes | 5 | Yes | Yes |
| Greece | Yes | SB | Yes | 2 | Yes | SB | Yes | 2 | Yes | NA | NA | NA | NA | Yes |
| Hungary | Yes | SA | No | 5 | Yes | SA | Yes | 5 | Yes | SA | No | 4 | Yes | Yes |
| Ireland | Yes | SA | Yes | 8 | Yes | SB | Yes | 9 | Yes | NA | NA | NA | NA | Yes |
| Israel | Yes | SB | Yes | 6 | Yes | SB | Yes | 6 | Yes | NA | NA | NA | NA | Yes |
| Italy | Yes | SA | No | 4 | Yes | SA | Yes | 4 | Yes | NA | NA | NA | NA | Yes |
| Kosovo | Yes | SA | Yes | 4 | Yes | SA | Yes | 5 | Yes | NA | NA | NA | NA | No |
| Latvia | NA | NA | NA | NA | NA | SA | Yes | 5 | Yes | NA | NA | NA | NA | No |
| Lithuania | Yes | SB | No | 4 | Yes | SA | Yes | 4 | Yes | NA | NA | NA | NA | No |
| Luxembourg | Yes | SB | No | NP | NP | SB | Yes | NP | NP | NA | NA | NA | NP | No |
| Malta | Yes | SA | Yes | 5 | Yes | SB | Yes | 5 | Yes | SB | Yes | 5 | Yes | Yes |
| Netherlands | Yes | SA | Yes | 5 | No | SB | Yes | 6 | Yes | NA | NA | NA | NA | Yes |
| Norway | Yes | SA | Yes | 5 | Yes | SB | Yes | 7 | Yes | NA | NA | NA | NA | Yes |
| Poland | Yes | SA | Yes | 4 | Yes | SA | Yes | 5 | Yes | NA | NA | NA | NA | Yes |
| Portugal | Yes | NA | NA | NA | NA | SA | Yes | 5 | Yes | NA | NA | NA | NA | No |
| Republic of Northern Macedonia | Yes | SB | Yes | NP | Yes | SB | Yes | NP | Yes | NA | NA | NA | NA | Yes |
| Romania | Yes | SA | Yes | 4 | Yes | SA | Yes | 5 | Yes | NA | NA | NA | NA | Yes |
| Serbia | Yes | SA | Yes | 4 | Yes | SA | Yes | 4 | Yes | SA | No | 3 | Yes | No |
| Slovakia | Yes | SA | No | 4 | Yes | SA | Yes | 4 | Yes | NA | NA | NA | NA | No |
| Slovenia | Yes | SA | Yes | 5 | Yes | SA | Yes | 6 | Yes | NA | NA | NA | NA | Yes |
| Spain | NA | SA | No | 4 | Yes | NA | NA | NA | NA | SA | Yes | 4 | Yes | No |
| Sweden | Yes | SA | Yes | 5 | Yes | SA | Yes | 5 | Yes | SA | Yes | 8 | Yes | Yes |
| Switzerland | Yes | SA | No | 4 | No | SA | Yes | 6 | Yes | NA | NA | NA | NA | Yes |
| Turkey | No | SA | Yes | 5 | Yes | SA | Yes | 5 | Yes | NA | NA | NA | NA | Yes |
| Ukraine | Yes | SA | No | NP | No | SA | No | NP | No | NA | NA | NA | NA | Yes |
| United Kingdom | No | SA | Yes | 9 | Yes | SB | Yes | 9 | Yes | NA | NA | NA | NA | Yes |

[a] Question only referring to medical doctors.

CM, Clinical Microbiology; ID, Infectious Diseases; IPC, Infection Prevention and Control; SA, stand-alone; SB, sub-specialty; MD, medical doctor; PG, postgraduate; NA, not applicable; NP, answer not provided/I do not know; Other, no acknowledgment as official specialty/sub-specialty, but existence of specific courses that allow some people to act as IPC professionals.

**eTable 5. Organization of AMS/IPC hospital activities at national level across Europe**

| Country | Collaboration | | AMS programmes | | | IPC programmes | | |
|---|---|---|---|---|---|---|---|---|
| | AMS and IPC activities under the same dpt/team /leader | Routine interactions between IPC and AMS professionals | National guidance or requirements on AMS implementation | Nationals staffing standards for AMS teams | National requirements for formal PG training to become an AMS team member[a] | National guidance or requirements on IPC implementation | Nationals staffing standards for IPC teams | National requirements for formal PG training to become an IPC team member[a] |
| Albania | Yes | NA | No | No | No | No | No | No |
| Austria | No | Yes | Only guidance | No | No | Yes, both | Yes | Yes |
| Azerbaijan | No | No | Only guidance | No | No | Only guidance | No | Yes |
| Belgium | No | Yes | Yes, both | Yes | No | Yes, both | Yes | Yes |
| Bulgaria | Yes | Yes | Only guidance | No | Yes | Yes, both | Yes | Yes |
| Croatia | Yes | Yes | No | No | No | Yes, both | Yes | Yes |
| Czech Republic | No | Yes | Only guidance | NP | No | Yes, both | Yes | Yes |
| Cyprus | No | NA | No | No | No | Only guidance | Yes | No |
| Denmark | No | NA | Yes, both | No | No | Yes, both | No | No |
| Estonia | Yes | NA | No | No | No | No | No | No |
| Finland | Yes | Yes | Only guidance | Yes | No | Yes, both | Yes | No |
| France | No | Yes | Only requirements | Yes | Yes | Only guidance | Yes | Yes |
| Germany | No | Yes | Only guidance | Yes | No | Yes, both | Yes | Yes |
| Greece | Yes | Yes | Only guidance | No | No | Only guidance | Yes | No |
| Hungary | Yes | Yes | Only guidance | Yes | No | Yes, both | Yes | Yes |
| Ireland | Yes | Yes | Yes, both | Yes | No | Yes, both | Yes | No |
| Israel | No | Yes | Yes, both | No | No | Yes, both | Yes | No |
| Italy | No | Yes | Only guidance | No | No | Only guidance | No | No |
| Kosovo | No | No | Only guidance | No | No | Yes, both | Yes | No |
| Latvia | Yes | NA | No | Yes | No | Yes, both | Yes | No |
| Lithuania | No | Yes | No | No | No | Yes, both | Yes | No |
| Luxembourg | Yes | Yes | Only guidance | No | No | Yes, both | No | No |
| Malta | Yes | Yes | No | No | No | Only guidance | No | Yes |
| Netherlands | No | Yes | Yes, both | Yes | No | Yes, both | Yes | No |
| Norway | Yes | Yes | Yes, both | No | No | Only guidance | No | No |
| Poland | Yes | Yes | Yes, both | No | No | No | Yes | No |
| Portugal | Yes | Yes | Only guidance | No | No | Only guidance | Yes | No |
| Republic of Northern Macedonia | Yes | Yes | Yes, both | No | No | Yes, both | No | No |
| Romania | No | No | No | No | No | Yes, both | Yes | No |
| Serbia | Yes | Yes | Only guidance | Yes | No | Only guidance | Yes | Yes |
| Slovakia | No | NA | NP | No | No | NP | No | No |
| Slovenia | No | Yes | Yes, both | No | No | Yes, both | Yes | No |
| Spain | No | Yes | Yes, both | No | No | No | No | Yes |
| Sweden | No | NA | Only guidance | No | No | Yes, both | No | Yes |
| Switzerland | Yes | Yes | Only guidance | No | No | No | No | No |
| Turkey | Yes | Yes | Only guidance | No | No | Yes, both | Yes | No |
| Ukraine | NP | Yes | Yes, both | No | Yes | Yes, both | NP | NP |
| United Kingdom | Yes | Yes | Yes, both | No | No | Yes, both | Yes | No |

[a] Question only referring to medical doctors.

AMS, Antimicrobial Stewardship; IPC, Infection Prevention and Control; DPT, department;Only PG, postgraduate; NA, not applicable; NP, answer not provided/I do not know.

**eTable 6. Web links to national guidance and requirements documents on AMS implementations in hospitals**

| Country | URL |
|---|---|
| Albania | Documents under development |
| Austria | Documents under development |
| Azerbaijan | NP |
| Belgium | http://overlegorganen.gezondheid.belgie.be/sites/default/files/documents/nationale_raad_voor_ziekenhuisvoorzieningen/2007_01_11_-_nrzv_d_286-2_nl.pdf; http://consultativebodies.health.belgium.be/sites/default/files/documents/2012_guide_belge_traitement_anti_infectieux_pratique_ambulatoire_antibioticagids_fr.pdf http://organesdeconcertation.sante.belgique.be/sites/default/files/documents/bapcoc_guidelineshospi_2017_sbimc-bvikm_fr_v1.pdf |
| Bulgaria | Documents under development |
| Croatia | Documents under development |
| Czech Republic | http://www.szu.cz/narodni-antibioticky-program |
| Cyprus | NP |
| Denmark | https://sum.dk/~/media/Filer%20-%20Publikationer_i_pdf/2017/Antibiotika-handlingsplan-frem-mod-2020/DK-Handlingsplan-05072017.pdf |
| Estonia | NP |
| Finland | https://stm.fi/julkaisu?pubid=URN:ISBN:978-952-00-3955-4 |
| France | https://www.has-sante.fr/portail/upload/docs/application/pdf/2016-04/2016_has_grille_de_recueil__icatb_2.pdf This indicator is currently being reevaluated by the national health agency |
| Germany | http://www.awmf.org/uploads/tx_szleitlinien/092-001l_S3_Antibiotika_Anwendung_im_Krankenhaus_2013-verlaengert.pdf; www.rki.de/DE/Content/Kommissi1n/ART/ART_node.html |
| Greece | NP |
| Hungary | www.oek.hu/oekfile.pl?fid=6619 |
| Ireland | NP |
| Israel | NP |
| Italy | http://www.salute.gov.it/portale/documentazi1/p6_2_2_1.jsp?lingua=italiano&id=2660 |
| Kosovo | NP |
| Latvia | Documents under development |
| Lithuania | NP |
| Luxembourg | http://www.sante.public.lu/fr/index.php |
| Malta | NP |
| Netherlands | www.ateams.nl |
| Norway | https://www.regjeringen.no/contentassets/915655269bc04a47928fce917e4b25f5/handlingsplan-antibiotikaresistens.pdf; https://www.antibiotika.no/antibiotikastyringsprogram-2/ |
| Poland | NP |
| Portugal | https://www.dgs.pt/programa-nacional-de-controlo-da-infeccao/despachos.aspx |
| Republic of Northern Macedonia | NP |
| Romania | https://www.srm.ro/pdf/protoco_MRSA_final%20mediere%20(2).pdf; http://www.srm.ro/pdf/ghid%20carbapenemaze_final_23.09.2015.pdf |
| Serbia | NP |
| Slovakia | NP |
| Slovenia | http://www.pisrs.si/Pis.web/pregledPredpisa?id=PRAV10625 http://www.pisrs.si/Pis.web/pregledPredpisa?id=PRAV2033 |
| Spain | https://www.sciencedirect.com/science/article/pii/S0213005X11003259?via%3Dihub http://www.resistenciaantibioticos.es/es/publicaciones/programas-de-optimizacion-del-uso-de-antibioticos-proa |
| Sweden | http://strama.se |
| Switzerland | Documents under development |
| Turkey | ttp://www.akilciilac.gov.tr |
| Ukraine | NP |
| United Kingdom | https://www.gov.uk/government/publications/the-health-and-social-care-act-2008-code-of-practice-on-the-prevention-and-control-of-infections-and-related-guidance#history; https://www.nice.org.uk/guidance/ng15 |

AMS, Antimicrobial Stewardship; URL, Uniform Resource Locator; NP, not provided (or absent).

**eTable 7. Details on national staffing standards for AMS hospital-based activities**

| Country | AMS |
|---|---|
| Albania | NP |
| Austria | NP |
| Azerbaijan | NP |
| Belgium | Minimal staff in hospital with minimum 150 beds: 1 FTE hospital hygiene nurse and 0.5 FTE hospital hygienist . http://overlegorganen.gezondheid.belgie.be/sites/default/files/documents/belgische_commissie_voor_de_coordinatie_van_het_antibioticabeleid/15174562.pdf |
| Bulgaria | NP |
| Croatia | NP |
| Czech Republic | NP |
| Cyprus | NP |
| Denmark | NP |
| Estonia | NP |
| Finland | NP |
| France | The appointed person should have the following dedicated time for AMS activities (not enforced in practice usually): 0,1 FTE / 400 rehabilitation or long-term care beds and 0,3 FTE / 400 acute care bedshttps://www.has-sante.fr/portail/upload/docs/application/pdf/2016-04/2016_has_grille_de_recueil__icatb_2.pdf |
| Germany | At least 1 FTE per 500 acute care bed http://www.awmf.org/uploads/tx_szleitlinien/092-001l_S3_Antibiotika_Anwendung_im_Krankenhaus_2013-verlaengert.pdf) |
| Greece | NP |
| Hungary | http://njt.hu/cgi_bin/njt_doc.cgi?docid=124091 |
| Ireland | https://www.hpsc.ie/a-z/microbiologyantimicrobialresistance/infectioncontrolandhai/guidelines/File,4116,en.pdf https://www.hiqa.ie/sites/default/files/2017-01/Antimicrobial-Stewardship-Review.pd |
| Israel | NP |
| Italy | NP |
| Kosovo | NP |
| Latvia | 1 ID specialist and 2 nurses to 500 acute care beds |
| Lithuania | NP |
| Luxembourg | NP |
| Malta | NP |
| Netherlands | www.swab.nl |
| Norway | NP |
| Poland | NP |
| Portugal | NP |
| Republic of Northern Macedonia | NP |
| Romania | NP |
| Serbia | NP |
| Slovakia | NP |
| Slovenia | NP |
| Spain | NP |
| Sweden | NP |
| Switzerland | NP |
| Turkey | NP |
| Ukraine | NP |
| United Kingdom | NP |

AMS, Antimicrobial Stewardship; IPC, Infection Prevention and Control; FTE,: full-time equivalent; ID, Infectious Diseases; NP, not provided (or absent).

**eTable 8. Web links to national guidance and requirements documents on IPC implementations in hospitals**

| Country | URL |
|---|---|
| Albania | NP |
| Austria | https://www.sozialministerium.at/site/Gesundheit/Gesundheitssystem/Gesundheitssystem_Qualitaetssicherung/Qualitaetsstandards/QS_Krankenhaushygiene_nbsp_Qualitaetsstandard_Organisation_und_Strategie_der_Krankenhaus_Hygiene |
| Azerbaijan | NP |
| Belgium | http://www.ejustice.just.fgov.be/cgi_loi/change_lg.pl?language=nl&la=N&table_name=wet&cn=2007042667; http://overlegorganen.gezondheid.belgie.be/sites/default/files/documents/belgische_commissie_voor_de_coordinatie_van_het_antibioticabeleid/15174562.pdf |
| Bulgaria | https://www.mh.government.bg/media/filer_public/2015/11/18/prevenciq-control-vutrebolnichni-infekcii.pdf |
| Croatia | NP |
| Czech Republic | https://www.mzcr.cz/obsah/program-prevence-a-kontroly-infekci-v-zdravotnickych-zarizenich_2917_5.html |
| Cyprus | https://www.moh.gov.cy/moh/moh.nsf/All/6B4121829D8502A5C2257C210026E74C |
| Denmark | https://www.ssi.dk/ |
| Estonia | NP |
| Finland | https://thl.fi/fi/web/infektiotaudit/ohjeet-ja-saadokset/saadokset/kansalliset/uusi-tartuntatautilaki-1.3.2017-alkaen-muutoksia-aiempaan/hoitoon-liittyvien-infektioiden-seuranta-ja-torjuntavelvoitteet |
| France | http://nosobase.chu-lyon.fr/Reglementation/2000/Circulaire/291200.pdf http://solidarites-sante.gouv.fr/IMG/pdf/2015_202to.pdf; http://solidarites-sante.gouv.fr/IMG/pdf/infections_nosocomiales_-_annexe_4_-_indicateur_optionnel.pdf |
| Germany | https://www.rki.de/DE/Content/Infekt/Krankenhaushygiene/Kommission/kommission_node.html; https://www.rki.de/DE/Content/Kommissi1n/KRINKO/krinko_node.html |
| Greece | http://www.eeel.gr/articlefiles/nomothesia/fek_388_2013_peri_enl.pdf |
| Hungary | http://njt.hu/cgi_bin/njt_doc.cgi?docid=1240; https://net.jogtar.hu/jogszabaly?docid=A0900020.EUM |
| Ireland | https://www.hpsc.ie/a-z/microbiologyantimicrobialresistance/infectioncontrolandhai/guidelines/File,4116,en.pdf; https://www.hiqa.ie/sites/default/files/2017-05/2017-HIQA-National-Standards-Healthcare-Association-Infections.pdf |
| Israel | NP |
| Italy | http://www.salute.gov.it/imgs/C_17_pagineAree_4621_listaFile_itemName_0_file.pdf |
| Kosovo | NP |
| Latvia | http://www.vi.gov.lv/lv/veselibas-aprupe/arstniecibas-iestades/info-arstn-iest-par-higieniska-un-pretepidemiska-rezima-plana-izstradi |
| Lithuania | NP |
| Luxembourg | NP |
| Malta | NP |
| Netherlands | NP |
| Norway | https://lovdata.no/dokument/SF/forskrift/2005-06-17-610?q=smittevern |
| Poland | NP |
| Portugal | https://www.dgs.pt/programa-nacional-de-controlo-da-infeccao/despachos.aspx |
| Republic of Northern Macedonia | NP |
| Romania | http://www.oamr.ro/wp-content/uploads/2016/09/Ord-MS-1101-2016.pdf |
| Serbia | NP |
| Slovakia | NP |
| Slovenia | http://www.uradni-list.si/1/objava.jsp?urlid=200633&stevilka=1348 http://www.uradni-list.si/1/objava.jsp?urlid=199974&stevilka=3597; http://www.uradni-list.si/1/objava.jsp?urlid=200692&stevilka=3969 |
| Spain | NP |
| Sweden | http://www.socialstyrelsen.se/english https://www.vardhandboken.se/om/om-vardhandboken/in-english/ https://www.folkhalsomyndigheten.se/the-public-health-agency-of-sweden/ |
| Switzerland | NP |
| Turkey | http://www.ttb.org.tr/mevzuat/index.php?option=com_content&view=article&id=333:yatakli-tedavkurumlari-enfekson-kontrol-yetmel&catid=2:ymelik&Itemid=33 |
| Ukraine | NP |
| United Kingdom | https://www.nice.org.uk/guidance/ph36; https://www.gov.uk/government/publications/review-of-the-infection-prevention-and-control-nurse-workforce |

IPC, Infection Prevention and Control; URL, Uniform Resource Locator; NP, not provided (or absent).

**eTable 9. Details on national staffing standards for IPC hospital-based activities**

| Country | IPC |
|---|---|
| Albania | NP |
| Austria | https://www.bmgf.gv.at/home/Gesundheit/Gesundheitssystem_Qualitaetssicherung/Qualitaetsstandards/QS_Krankenhaushygiene_nbsp_ https://www.sozialministerium.at/site/Gesundheit/Gesundheitssystem/Gesundheitssystem_Qualitaetssicherung/Qualitaetsstandards/-QS_Krankenhaushygiene_nbsp_Qualitaetsstandard_Organisation_und_Strategie_der_Kranken 1 FTE IPC-MD/400-800 beds and 1 FTE nurse/150-400 beds depending on type of hospital |
| Azerbaijan | NP |
| Belgium | 1 FTE MD-IPC/1000 beds and 1 FTE nurse-IPC/400 beds |
| Bulgaria | 1 FTE CM specialist plus 1 FTE IPC nurse per 500 care beds for the IPC team |
| Croatia | 1 FTE nurse /250 beds |
| Czech Republic | Minimal personal standards for hospital hygiene departments 1000 beds: 1 MD + 2 nonMDs every other 300 beds: + 1 nonMD |
| Cyprus | here is a FTE nurse for every 250 acute care beds |
| Denmark | NP |
| Estonia | NP |
| Finland | NP |
| France | 1 FTE IPC nurse for 400 beds 1 FTE IPC MD or PharmD for 800 beds http://nosobase.chu-lyon.fr/Reglementation/2000/Circulaire/291200.pdf |
| Germany | At least 1 FTE per 400 acute care bed, also depending on the region http://www.awmf.org/uploads/tx_szleitlinien/092-001l_S3_Antibiotika_Anwendung_im_Krankenhaus_2013-verlaengert.pdf) |
| Greece | http://www.eeel.gr/articlefiles/nomothesia/fek_388_2013_peri_enl.pdf |
| Hungary | http://njt.hu/cgi_bin/njt_doc.cgi?docid=124091 |
| Ireland | 1 full-time IPC nurse for 150-200 patients https://www.hpsc.ie/a-z/microbiologyantimicrobialresistance/infectioncontrolandhai/guidelines/File,4116,en.pdf |
| Israel | NP |
| Italy | NP |
| Kosovo | 1 IPC nurse in 500 beds |
| Latvia | 1 ID and 2 nurses to 500 acute care beds. |
| Lithuania | 1 IPC doctor for 400 hospital beds, or 100 000 ambulatory consultations in polyclinic 1 IPC nurse for 100 hospital beds, or 50 000-100 000 ambulatory consultations in polyclinic https://e-seimas.lrs.lt/portal/legalAct/lt/TAD/TAIS.332026 |
| Luxembourg | NP |
| Malta | NP |
| Netherlands | 1 nurse per 5000 admissions, 1 MD per 25000 admissions www.igj.nl |
| Norway | NP |
| Poland | Just for nurses: 1 FTE of IPC nurse per 200 beds |
| Portugal | 1 FTE nurse per 250 beds 1 FTE physicians per hospital with more than 250 beds |
| Republic of Northern Macedonia | NP |
| Romania | 1 epidemiologist and 1 ID specialist for every 400 beds and 1 nurse for every 250 beds http://www.oamr.ro/wp-content/uploads/2016/09/Ord-MS-1101-2016.pdf |
| Serbia | NP |
| Slovakia | NP |
| Slovenia | NP |
| Spain | NP |
| Sweden | NP |
| Switzerland | NP |
| Turkey | Each 150 occupied beds, 1 FTE IPC nurse is required; http://www.resmigazete.gov.tr/eskiler/2005/08/20050811-6.htm |
| Ukraine | NP |
| United Kingdom | https://www.gov.uk/government/uploads/system/uploads/attachment_data/file/449049/Code_of_practice_280715_acc.pdf |

AMS, Antimicrobial Stewardship; IPC, Infection Prevention and Control; FTE, full-time equivalent; MD, Medical Doctor; CM, Clinical Microbiology; ID, Infectious Diseases; NP, not provided (or absent).

**eTable 10. Educational national requirements regarding AMS and IPC during postgraduate training across Europe (all questions only referring to medical doctors)**

| Country | Antimicrobial stewardship | | | | | | Infection Prevention and Control | | | | | |
|---|---|---|---|---|---|---|---|---|---|---|---|---|
| | Mandatory formal training on AMS implementation during training | | | Mandatory involvement in AMS activities during training | | | Mandatory formal training on IPC implementation during training | | | Mandatory involvement in IPC activities during training | | |
| *Trainee category* | CM | ID | IPC | CM | ID | IPC | CM | ID | IPC | CM | ID | IPC |
| Albania | No | No | NA | No | No | NA | No | No | NA | No | No | NA |
| Austria | Yes | Yes | Yes | No | No | No | Yes | No | Yes | Yes | No | Yes |
| Azerbaijan | Yes | No | NP | Yes | No | NP | No | No | NP | No | No | NP |
| Belgium | Yes | Yes | Yes | Yes | Yes | Yes | Yes | Yes | Yes | Yes | Yes | Yes |
| Bulgaria | Yes | No | Yes | Yes | Yes | Yes | Yes | No | Yes | Yes | NA | Yes |
| Croatia | No | No | No | No | Yes | No | Yes | No | Yes | Yes | Yes | Yes |
| Czech Republic | Yes | No | No | Yes | No | No | No | No | Yes | No | No | Yes |
| Cyprus | No | NP | NA | No | No | NA | No | NP | NA | No | No | NA |
| Denmark | No | No | NA | No | No | NA | No | No | NA | No | No | NA |
| Estonia | No | No | No | NP | Yes | Yes | No | No | No | No | Yes | Yes |
| Finland | No | No | No | No | No | No | No | No | No | No | Yes | NP |
| France | No | Yes | Yes | No | Yes | No | No | No | Yes | No | No | Yes |
| Germany | No | No | No | No | No | No | No | No | Yes | Yes | Yes | Yes |
| Greece | No | Yes | NA | No | No | NA | Yes | Yes | NA | Yes | Yes | NA |
| Hungary | No | No | No | Yes | Yes | Yes | Yes | Yes | No | Yes | Yes | Yes |
| Ireland | Yes | Yes | NA | Yes | Yes | NA | Yes | No | NA | Yes | Yes | NA |
| Israel | No | Yes | NA | No | Yes | NA | No | No | NA | No | No | NA |
| Italy | No | No | NA | No | No | NA | No | No | NA | No | No | NA |
| Kosovo | No | No | NA | No | No | NA | No | No | NA | No | No | NA |
| Latvia | NA | No | NA | NA | Yes | NA | NA | Yes | NA | NA | Yes | NA |
| Lithuania | No | No | NA | No | No | NA | No | No | NA | NP | NP | NA |
| Luxembourg | No | No | NA | No | No | No | NP | NP | NP | NP | NP | NA |
| Malta | NP | NP | NP | Yes | No | Yes | NP | NP | NP | Yes | No | Yes |
| Netherlands | No | No | NA | Yes | Yes | NA | Yes | No | NA | Yes | No | NA |
| Norway | No | No | NA | No | No | NA | Yes | Yes | NA | No | No | NA |
| Poland | Yes | No | NA | Yes | No | NA | Yes | No | NA | Yes | No | NA |
| Portugal | NA | No | NA | NA | No | NA | NA | No | NA | NA | No | NA |
| Republic of Northern Macedonia | NP | NP | NA | NP | NP | NA | Yes | Yes | NA | Yes | Yes | NA |
| Romania | No | No | NA | No | No | NA | Yes | Yes | NA | Yes | Yes | NA |
| Serbia | Yes | Yes | Yes | Yes | Yes | Yes | No | Yes | Yes | No | Yes | Yes |
| Slovakia | No | No | NA | No | No | NA | No | No | NA | No | No | NA |
| Slovenia | No | No | NA | No | Yes | NA | Yes | Yes | NA | No | No | NA |
| Spain | No | NA | No | No | NA | No | No | NA | Yes | No | NA | Yes |
| Sweden | No | No | No | No | No | No | Yes | Yes | Yes | Yes | Yes | Yes |
| Switzerland | No | No | NA | No | No | No | No | Yes | No | No | No | NA |
| Turkey | No | No | NA | No | No | NA | No | No | NA | No | Yes | NA |
| Ukraine | Yes | Yes | NA | Yes | Yes | NA | Yes | Yes | NA | Yes | Yes | NA |
| United Kingdom | No | No | NA | No | No | NA | Yes | Yes | NA | Yes | Yes | NA |

AMS, Antimicrobial Stewardship; IPC, Infection Prevention and Control; PG, postgraduate; CM, Clinical Microbiology; ID, Infectious Diseases; IPC, Infection Prevention and Control; NA, not applicable; NP, answer not provided/I do not know.

**eTable 11. Professionals usually in charge of running AMS and IPC activities in European hospitals**

| Country | Antimicrobial stewardship | | | | | Infection Prevention and Control | | | | |
|---|---|---|---|---|---|---|---|---|---|---|
| *Category* | CM | ID | Pharmacist | Nurse | Other | CM | ID | Pharmacist | Nurse | Other |
| Albania | X | X | X | | | X | X | | X | |
| Austria | X | X | X | | X | X | X | | X | X |
| Azerbaijan | X | | | | | X | X | | | |
| Belgium | X | X | X | | X | X | | | X | X |
| Bulgaria | X | | X | | | X | | | X | X |
| Croatia | X | X | | | X | X | X | | X | X |
| Czech Republic | X | X | | | | | | | X | X |
| Cyprus | | | | | X | | X | | X | |
| Denmark | X | X | X | | | X | X | | X | |
| Estonia | | X | | | | | X | | X | |
| Finland | | X | | | | X | X | | X | |
| France | X | X | X | | X | X | | X | X | X |
| Germany | X | X | X | X | X | X | | | | X |
| Greece | | X | X | | | X | X | X | X | X |
| Hungary | | X | | | | | X | | X | X |
| Ireland | X | X | X | X | | X | | | X | X |
| Israel | | X | | | | | X | | X | |
| Italy | | X | X | | | | | | X | X |
| Kosovo | X | X | X | | | X | X | | X | |
| Latvia | | X | X | X | | | | | X | |
| Lithuania | X | | X | | X | | | | | |
| Luxembourg | X | X | X | | | X | X | X | | |
| Malta | X | X | X | | | | | | X | |
| Netherlands | X | X | X | | | X | | | X | |
| Norway | X | X | X | X | | X | X | | X | |
| Poland | X | X | X | | | X | X | | X | |
| Portugal | | X | X | X | | | X | | X | |
| Republic of Northern Macedonia | | | | X | X | | | | X | X |
| Romania | | X | | | X | | X | | X | X |
| Serbia | | X | X | | | | X | X | | |
| Slovakia | | | X | | | | X | | | |
| Slovenia | X | X | X | | | X | X | | X | X |
| Spain | X | | X | | X | X | | | X | X |
| Sweden | X | X | X | X | | X | X | | X | X |
| Switzerland | | X | | | | | X | | X | |
| Turkey | X | X | | | | X | X | | X | |
| Ukraine | X | | | X | X | | | | X | X |
| United Kingdom | X | X | X | | | X | X | | X | |

CM, Clinical Microbiology; ID, Infectious Diseases

**eTable 12. Other aspects of usual practice of professionals involved in AMS/IPC in European countries**

| Country | Miscellaneous | | |
|---|---|---|---|
| | CM & ID specialists work exclusively in hospitals (only referring to MD) | CM usually do clinical rounds on hospital wards | IPC specialists usually do clinical rounds on hospital wards |
| Albania | No | No | NA |
| Austria | No | Yes | Yes |
| Azerbaijan | Yes for both | No | Yes |
| Belgium | No | Yes | Yes |
| Bulgaria | Yes for both | Yes | Yes |
| Croatia | Only ID | Yes | Yes |
| Czech Republic | Yes for both | Yes | Yes |
| Cyprus | No | No | NA |
| Denmark | Yes for both | No | NA |
| Estonia | No | No | Yes |
| Finland | Yes for both | No | Yes |
| France | No | No | Yes |
| Germany | No | Yes | Yes |
| Greece | Only ID | No | NA |
| Hungary | Only ID | No | Yes |
| Ireland | Yes for both | Yes | NA |
| Israel | Yes for both | No | NA |
| Italy | No | No | NA |
| Kosovo | Only ID | Yes | NA |
| Latvia | No | NA | NA |
| Lithuania | Yes for both | Yes | NA |
| Luxembourg | Only ID | Yes | NA |
| Malta | Yes for both | Yes | No |
| Netherlands | Only ID | Yes | NA |
| Norway | Yes for both | Yes | NA |
| Poland | Only CM | Yes | NA |
| Portugal | No | NA | NA |
| Republic of Northern Macedonia | No | NP | NA |
| Romania | Only ID | No | NA |
| Serbia | Only ID | No | No |
| Slovakia | Only ID | Yes | NA |
| Slovenia | Only ID | No | NA |
| Spain | Only CM | No | No |
| Sweden | No | No | Yes |
| Switzerland | Yes for both | No | NA |
| Turkey | Yes for both | Yes | NA |
| Ukraine | No | Yes | NA |
| United Kingdom | Only ID | Yes | NA |

AMS, Antimicrobial Stewardship; IPC, Infection Prevention and Control; CM, Clinical Microbiology; ID, Infectious Diseases; MD, medical doctor; IPC, Infection Prevention and Control; NA, not applicable; NP, answer not provided/I do not know.
